# Homographically generated light sheets for the microscopy of large specimens

**DOI:** 10.1364/OL.43.000663

**Published:** 2018-02-06

**Authors:** Craig T. Russell, Eric J. Rees, Clemens F. Kaminski

**Affiliations:** Department of Chemical Engineering and Biotechnology, University of Cambridge, Cambridge CB2 3RA, UK

**Keywords:** (180.6900) Three-dimensional microscopy, (180.2520) Fluorescence microscopy

## Abstract

We compare the performance of linear and nonlinear methods for aligning the excitation and detection planes throughout volumes of large specimens in digitally scanned light sheet microscopy. An effective nonlinear method involves the registration of four corner extrema of the imaging volume *via* a projective transform. We show that this improves the light collection efficiency of the commonly used three-point affine registration by an average of 42% over a typical specimen volume. Accurate illumination/detection registration methods are now pertinent to biological research in view of current trends towards imaging large or expanded samples, at depth, with diffraction limited resolution.

Light sheet fluorescence microscopy is fast becoming the method of choice for imaging large volumetric samples. Whilst confocal techniques reject the out-of-focus signal excited by the illumination source through use of a pinhole, light sheet technology is a much more efficient optical sectioning method, since signals are exclusively generated in the plane defined by the thin sheet of illumination light.

As light sheet imaging is a wide-field technique, the temporal resolution is much higher than achievable via confocal scanning, and the photon dosage for generating an equally bright image is ∼2 orders of magnitude lower. This makes the method ideally suited for the imaging of live biological specimens [1]. Commercial and home-built [2] light sheet systems often use a cylindrical lens to convert a circular Gaussian laser beam into a thin sheet. Alternatively, galvanometric mirrors can be used to produce a light sheet by rapidly dithering a laser beam; this is referred to as digitally scanned light sheet microscopy (DSLM) [3]. Typically, a galvanometric mirror pair is used to sweep an incident laser beam through a scan lens which converts a beam angle to a beam position; within the observation volume, this acts to keep the sweeping beam parallel for a homogenous illumination and background [4]. However, the use of a scan lens can lead to registration errors of the light sheet with respect to the imaging plane, reducing contrast, and increasing background fluorescence. Thus, accurate registration is critical for the technique. In this Letter, we compare the performance of nonlinear and linear methods for registering excitation and imaging planes in a DSLM system. We introduce a novel homographic method and discuss its advantages in practical light sheet microscopy.

Aligning a digitally scanned light sheet to a detection plane requires the generation of control signals $V_{x}$, $V_{z}$ for the scanning mirrors to generate a light sheet in the x, y plane, which is translated in z (geometry as in Fig. 1

**Fig. 1.**
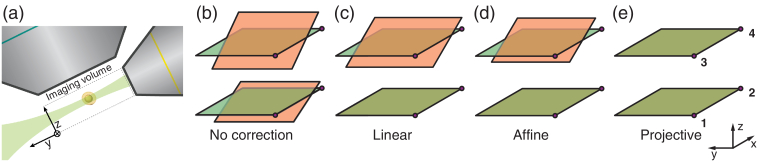
(a) Schematic of the light sheet optics using a large NA detection objective. The beam is scanned in x to create a virtual sheet. (b)–(e) For the best image quality, the illumination planes (shown as red when not perfectly registered to the illumination planes) must be registered to the detection planes (green). (c) The linear registration using coordinates (**1,2**) is sufficient to register one plane. The affine registration (d) is commonly used in DSLM systems and provides reasonable performance by registering three points, [typically (**1,2**) and the midpoint of (**3,4**)]. However, the projective registration (e) using four control points (**1**–**4**) provides superior performance due to decreased out-of-focus fluorescence.

). In two dimensions, the extrema in x define two edges of the imaging field of view (FOV) and a linear ramp in $V_{x}$ between these coordinates produces a virtual light sheet. The z-mirror extrema correspond to the top and bottom planes of the image stack. However, using linear ramping from a given starting point, only three of the four x and z extrema can be registered [5]. The fourth point is either discarded or, more typically, amalgamated with the third by an averaging of the available vertices in one of the planes. As illustrated in Fig. 1, this assumption then leads to a poorly registered illumination in the plane where the fourth coordinate was neglected, and greater background fluorescence in 3D imaging.

The stack of illumination planes used in a 3D observation can be better matched to the detection planes by registering four corners of the available excitation 3D-FOV, using a projective transform. Projective transforms can map any quadrilateral onto any other, whereas an affine transformation can only register three points. Higher order corrections could also be used; one example is an n-point correction using b-splines; however, this is computationally expensive. Such elastic transforms require a larger number of correspondences and, therefore, are likely to incur compounded errors.

A calibration experiment provides the control signals ($V_{x_{i}}$, $V_{z_{i}}$) for i=1 to 4, needed to register the illumination to the four extrema of the imaging volume, ($x_{i}$, $z_{i}$). In a projective transform of r=(x,z), we generate the augmented vector $\overset{˜}{r}=(x,z,1)$ and then apply a linear transform to obtain ${\overset{˜}{r}}^{\prime }=H\overset{˜}{r}$. This is followed by descaling to obtain the transformed vector:

$$r^{\prime }=\left(\frac{{\overset{˜}{r}}_{1}^{\prime }}{{\overset{˜}{r}}_{3}^{\prime }}\frac{{\overset{˜}{r}}_{2}^{\prime }}{{\overset{˜}{r}}_{3}^{\prime }}\right).$$ (1)

A projective transform of a plane can be exactly defined by four projected points, unless any three are collinear. Now the calibration experiment identifies four (noncollinear) extrema of the imaging volume, and it is possible to combine the augmented form of three of these positions to produce the fourth, such that

$$\left(\begin{array}{ccc} x_{1} & x_{2} & x_{3}\\ z_{1} & z_{2} & z_{3}\\ 1 & 1 & 1 \end{array}\right)\;\left(\begin{array}{c} \lambda \\ \mu \\ \nu \end{array}\right)=\left(\begin{array}{c} x_{4}\\ z_{4}\\ 1 \end{array}\right)\;,$$ (2)

where λ,μ and ν are constants. After solving for λ,μ and ν, the matrix A can be constructed:

$$A=\left(\begin{array}{ccc} \lambda x_{1} & \mu x_{2} & \nu x_{3}\\ \lambda z_{1} & \mu z_{2} & \nu z_{3}\\ \lambda & \mu & \nu \end{array}\right).$$ (3)

The matrix A maps basis vectors to the specific points chosen for registration:

$$\begin{array}{c} A{\left(100\right)}^{T}\mapsto \lambda {\left(x_{1},z_{1},1\right)}^{T}\\ A{\left(010\right)}^{T}\mapsto \mu {\left(x_{2},z_{2},1\right)}^{T}\\ A{\left(001\right)}^{T}\mapsto \nu {\left(x_{3},z_{3},1\right)}^{T}\\ A{\left(111\right)}^{T}\mapsto {\left(x_{4},z_{4},1\right)}^{T}. \end{array}$$

Here A acts on the basis column vectors which are written here as transposed row vectors with the superscript T, and the resulting augmented coordinates descale to the registration coordinates via Eq. (1). Since A maps basis vectors to augmented positions, $A^{-1}$ decomposes an augmented position into basis vectors.

Now the calibration experiment provides control signals ($V_{x_{i}}$, $V_{z_{i}}$) which can be transformed to augmented vectors and treated in the same way. Specifically, $a(V_{x_{1}},V_{z_{1}},1)+b(V_{x_{2}},V_{z_{2}},1)+c(V_{x_{3}},V_{z_{3}},1)=(V_{x_{4}},V_{z_{4}},1)$ for constants a,b, and c so that

$$\left(\begin{array}{c} a\\ b\\ c \end{array}\right)={\left(\begin{array}{ccc} V_{x_{1}} & V_{x_{2}} & V_{x_{3}}\\ V_{z_{1}} & V_{z_{2}} & V_{z_{3}}\\ 1 & 1 & 1 \end{array}\right)}^{-1}\;\left(\begin{array}{c} V_{x_{4}}\\ V_{x_{4}}\\ 1 \end{array}\right).$$ (4)

The matrix B can be created in the same way as A:

$$B=\left(\begin{array}{ccc} ax_{1} & bx_{2} & cx_{3}\\ az_{1} & bz_{2} & cz_{3}\\ a & b & c \end{array}\right).$$ (5)

B maps from basis vectors to augmented signals, so that $B{\left(111\right)}^{T}={\left(V_{x_{4}},V_{z_{4}},1\right)}^{T}$. To compute the projective transform of an illumination position r=(x,z) to the required control signal $V=(V_{x},V_{z})$, we simply need to convert the augmented position to basis vectors using $A^{-1}\overset{˜}{r}$, and the basis vectors to control signals using B followed by dehomogenization using Eq. (1). It is useful to use the homography matrix $H={\mathrm{BA}}^{-1}$, so that $\overset{˜}{V}=H\overset{˜}{r}$, or

$$\left(\begin{array}{c} {\overset{˜}{V}}_{x}\\ {\overset{˜}{V}}_{z}\\ k \end{array}\right)=\left(\begin{array}{ccc} aV_{x_{1}} & bV_{x_{2}} & cV_{x_{3}}\\ aV_{z_{1}} & bV_{z_{2}} & cV_{z_{3}}\\ a & b & c \end{array}\right)\;{\left(\begin{array}{ccc} 0 & 1 & 0\\ -z_{1} & z_{2} & z_{3}\\ -1 & 1 & 1 \end{array}\right)}^{-1}\;\left(\begin{array}{c} x\\ z\\ 1 \end{array}\right)\;,$$ (6)

where the x range is normalized to run from $x_{1}=x_{3}=0$ to $x_{2}=x_{4}=1$, and $A^{-1}$ is heavily simplified by solving for λ,μ and ν. Finally,

$$\left(\begin{array}{c} V_{x}\\ V_{z} \end{array}\right)=\frac{1}{k}\;(\begin{array}{c} {\overset{˜}{V}}_{x}\\ {\overset{˜}{V}}_{z} \end{array})$$ (7)

rescales homogenous voltages to real output voltages. Therefore, non-extrema points can readily be interpolated to create signal trains; for higher-order corrections, point-wise generation would be necessary. All demonstrations of the four-point correction were performed on an [6] inverted Selective Plane Imaging Microscope (iSPIM). A (*Coherent Obis 561 nm*) laser was used as the beam source. A pair of galvanometric mirrors (*Cambridge Technology*) was used to produce 2D beam steering via an image relay; this approach is well established in scanning microscopy and is known to introduce ordinarily negligible field curvature. A telecentric scan lens (*A1 Scan Lens* from Nikon) was used to parallelize the scanning beam; a (10×0.3NA Nikon) water dipping objective was used for excitation and mounted at right angles to a (25×1.1NA) Nikon LWD water immersion objective. The fluorescence collected by the detection objective is then imaged onto a Hamamatsu sCMOS Orca Flash 4. A piezo scanner (*Physik Instrumente P-726 PIFOC high-load objective scanner*) was used to manually move the detection objective to match the detection focal plane to the excitation plane. The back aperture of the excitation objective was stopped down with an adjustable iris; the work presented in this Letter produced a light sheet of 8 μm with a confocal extension of 180 μm for a laser color of 561 nm.

To accurately measure the deviation solely caused by the scanning system, a camera (Thorlabs DCC1545M) was mounted directly after the scan lens and an attenuated beam was imaged directly onto the sensor. The full range of the scanning unit was studied by incrementing mirror control voltages ($V_{x}$, $V_{z}$) linearly and imaging the illumination beam in xz for each step, as shown in Fig. 2(a)

**Fig. 2.**
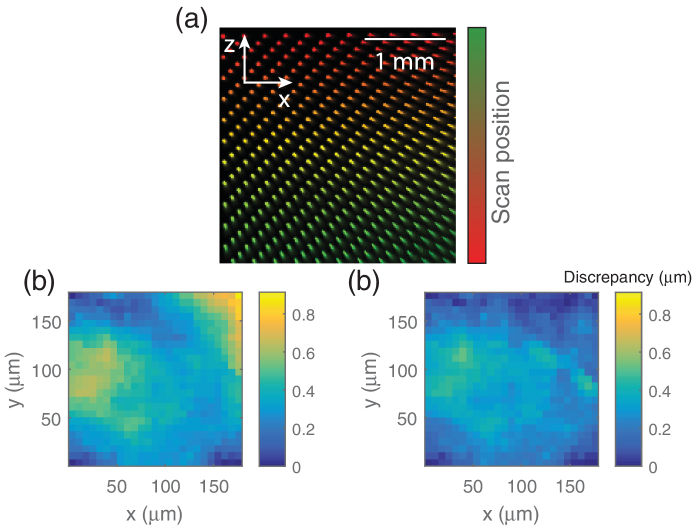
Scan lens characterization: (a) shows the illumination profile in the xz plane for 400 scan positions, with a three-point registration. Each of the beam positions in (a) was localized by fitting a 2D Gaussian. The identified positions, using (b) a three point and (c) a four point, show that the positional discrepancy of the three-point method is largely fixed by the four-point registration; mean average discrepancy values being reduced for the affine versus homographic correction of 0.3673 μm and 0.2494 μm, respectively, with standard deviation values also dropping from 0.1467 μm to 0.1133 μm.

. Each beam profile was fit with a 2D Gaussian to create a map of xz illumination positions corresponding to the constant steps in the scan lens. Figures 2(b) and 2(c) show the residual deviation from the desired positions when using a four-point and three-point registration, respectively. The four-point correction is more faithful to experimental values. The figure verifies that a four-point correction produces a more valid fitting for a beam scanned across a telecentric lens, with a more significant improvement becoming apparent when using a larger region of the scan lens.

In real samples for light sheet microscopy, a mismatch between the detection plane and the illumination plane can reduce image fidelity due to decreased illumination in the imaging plane, as well as excess background fluorescence. Figure 3

**Fig. 3.**
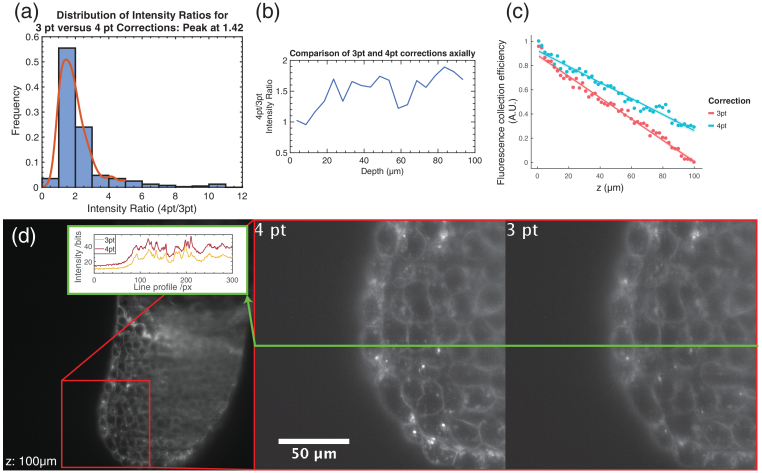
*In situ* characterization: (a), (b) ratios of intensity maxima of localized fluorescent bead images were compared in 3D observation volumes using three- and four-point corrections. (a) On average, a 42% increase in contrast was obtained for the four-point case, with benefits increasing as the depth increases (b). (c) Corresponding graph for a beam scanned through a dye solution. The four-point correction results in greater light capture efficiency over the three-point correction, which again becomes more significant with depth. Here the brightness of the beam’s image indicated how well it was registered to the imaging plane. (d) Transgenic zebrafish expressing mCherry:beta-actinCAAX. The contrast of features is substantially improved by the four-point registration, and the images afford greater detail and clarity, as shown by inset line profiles.

shows that the four-point registration largely eliminates this mismatch for real samples, including fluorescent beads, dyes, and a model organism.

In Figs. 3(a) and 3(b), fluorescent beads (TetraSpeck 100 nm Microspheres) were dispersed in 1.5% agarose at 1:1000 concentration and imaged using a three-point and a four-point registration. Each bead (from a total of ∼500) was localized in 3D, and its peak fluorescence intensity was compared in the four-point and three-point case, and was found to be, on average, 42% higher across the entire volume (512μm×512μm×100μm) for the four-point registration. For our Gaussian beam of 8 μm width (full-width at half-maximum), this corresponds to correcting a light sheet misregistration of about 4 μm on average. The experiment from Fig. 2 was repeated in the light sheet microscope using a dye solution for Fig. 3(c). The scanning beam was paused and iterated again through discrete positions in the imaging volume. Each record fluorescent dye image was characterized by a focus measure, obtained by finding the intensity maximum through the focus of the light sheet for each beam position. As expected, the mismatch between the light sheet and image plane increased more quickly with the imaging depth for the three-point correction than in the case of the four-point correction.

The advantages of using a four-point correction, finally, were then demonstrated in fluorescence images from a transgenic zebrafish sample, a model organism ubiquitous in light sheet imaging. The sample shown in Fig. 3(d) expressed an mCherry fluorescent protein construct (Beta-actin: mCherryCAAX) near the cellular membrane and was mounted in 1.2% agarose; imaging took place 4 h post-fertilization. The greater clarity of detail and contrast afforded by the four-point method is evident.

The registration between detection and illumination volumes [7] is becoming a greater challenge, as high-resolution light sheet methods are used to image ever larger and deeper samples [8–10]. Both increasing chip sizes in cameras [11,12] and imaging with large FOVs [13] create a need for effective registration methods that can avoid errors which would otherwise be introduced by using linear waveforms in light sheet microscopes. In addition, it is especially valuable to have excellent registration when rolling confocal slit scanning cameras are employed [14]: these systems achieve an anisotropic improvement in resolution and contrast, but suffer more severely from misalignment because out-of-focus light is blocked by the detection slit and can lead to dark, rather than merely blurred images.

In conclusion, we have demonstrated that, for iSPIM systems with digitally scanned light sheets, a four-point correction achieved by nonlinear waveform generation affords better correction than a three-point method (affine waveform generation) against errors introduced by beam scanning optics, offering a practical method that is conveniently implemented and requires minimal computational effort. In our iSPIM, the four-point correction was applied optomechanically to control the light sheet position, but a similar approach might be used to ensure well-registered sample positioning in systems where the sample is moved instead of the light sheet. There are other sources of light sheet displacement, such as refraction by texture in the specimen; however, these issues may need to be corrected on a specimen-wise basis, and their correction goes beyond the scope of this Letter.

**Software**: Examples and implementations of the methods described above are provided in both MATLAB and LabVIEW.

## References

[1] Huisken J. Swoger J. Bene F. D. Wittbrodt J. Stelzer E. H. K., Science 305, 1007 (2004). SCIEAS 0036-8075 10.1126/science.1100035 15310904

[2] Pitrone P. G. Schindelin J. Stuyvenberg L. Preibisch S. Weber M. Eliceiri K. W. Huisken J. Tomancak P., Nat. Methods 10, 598 (2013).1548-7091 10.1038/nmeth.2507 23749304 PMC7450513

[3] Keller P. J. Schmidt A. D. Santella A. Khairy K. Bao Z. Wittbrodt J. Stelzer E. H. K., Nat. Methods 7, 637 (2010).1548-7091 10.1038/nmeth.1476 20601950 PMC4418465

[4] Chen B. C. Legant W. R. Wang K. Shao L. Milkie D. E. Davidson M. W. Janetopoulos C. Wu X. S. Hammer J. A. Liu Z. English B. P., Science 346, 1257998 (2014). SCIEAS 0036-8075 10.1126/science.1257998 25342811 PMC4336192

[5] Zitová B. Flusser J., Image Vis. Comput. 21, 977 (2003). IVCODK 0262-8856 10.1016/S0262-8856(03)00137-9

[6] Chmielewski A. K. Kyrsting A. Mahou P. Wayland M. T. Muresan L. Evers J. F. Kaminski C. F., Sci. Rep. 5, 9385 (2015). SRCEC3 2045-2322 10.1038/srep09385 25893952 PMC4403519

[7] Royer L. A. Lemon W. C. Chhetri R. K. Wan Y. Coleman M. Myers E. W. Keller P. J., Nat. Biotechnol. 34, 1267 (2016). NABIF9 1087-0156 10.1038/nbt.3708 27798562

[8] Chen F. Tillberg P. W. Boyden E. S., Science 347, 543 (2015). SCIEAS 0036-8075 10.1126/science.1260088 25592419 PMC4312537

[9] Wang K. Sun W. Richie C. T. Harvey B. K. Betzig E. Ji N., Nat. Commun. 6, 7276 (2015). NCAOBW 2041-1723 10.1038/ncomms8276 26073070 PMC4490402

[10] Truong T. V. Supatto W. Koos D. S. Choi J. M. Fraser S. E., Nat. Methods 8, 757 (2011).1548-7091 10.1038/nmeth.1652 21765409

[11] Zheng G. Ou X. Yang C., Biomed. Opt. Express 5, 1 (2014). BOEICL 2156-7085 10.1364/BOE.5.000001 PMC3891323 24466471

[12] Brady D. J. Gehm M. E. Stack R. A. Marks D. L. Kittle D. S. Golish D. R. Vera E. M. Feller S. D., Nature 486, 386 (2012). NATUAS 0028-0836 10.1038/nature11150 22722199

[13] Sofroniew N. J. Flickinger D. King J. Svoboda K., eLife 5, e14472 (2016). ELIFA8 2050-084X 10.7554/eLife.14472 27300105 PMC4951199

[14] Baumgart E. Kubitscheck U., Opt. Express 20, 21805 (2012). OPEXFF 1094-4087 10.1364/OE.20.021805 23037300

